# Fine-Scale Genetic Structure in Finland

**DOI:** 10.1534/g3.117.300217

**Published:** 2017-08-29

**Authors:** Sini Kerminen, Aki S. Havulinna, Garrett Hellenthal, Alicia R. Martin, Antti-Pekka Sarin, Markus Perola, Aarno Palotie, Veikko Salomaa, Mark J. Daly, Samuli Ripatti, Matti Pirinen

**Affiliations:** * Institute for Molecular Medicine Finland, University of Helsinki, 00014, Finland; † National Institute for Health and Welfare, 00271 Helsinki, Finland; ‡ University College London Genetics Institute, University College London, WC1E 6BT, UK; § Analytic and Translational Genetics Unit, Massachusetts General Hospital, Boston, Massachusetts 02114; ** Program in Medical and Population Genetics, Broad Institute of Massachusetts Institute of Technology and Harvard, Cambridge, Massachusetts 02142; †† Stanley Center for Psychiatric Research, Broad Institute of Massachusetts Institute of Technology and Harvard, Cambridge, Massachusetts 02142; ‡‡ The Estonian Genome Center, University of Tartu, 51010, Estonia; §§ Psychiatric and Neurodevelopmental Genetics Unit, Department of Psychiatry, Massachusetts General Hospital, Boston, Massachusetts 02114; *** Department of Neurology, Massachusetts General Hospital, Boston, Massachusetts 02114; ††† Department of Public Health, University of Helsinki, 00014, Finland; ‡‡‡ Wellcome Trust Sanger Institute, Wellcome Trust Genome Campus, Hinxton CB10 1SA, UK; §§§ Helsinki Institute for Information Technology, University of Helsinki, 00014, Finland; ‡‡‡ Department of Mathematics and Statistics, University of Helsinki, 00014, Finland

**Keywords:** population genetics, population structure, haplotype sharing

## Abstract

Coupling dense genotype data with new computational methods offers unprecedented opportunities for individual-level ancestry estimation once geographically precisely defined reference data sets become available. We study such a reference data set for Finland containing 2376 such individuals from the FINRISK Study survey of 1997 both of whose parents were born close to each other. This sampling strategy focuses on the population structure present in Finland before the 1950s. By using the recent haplotype-based methods ChromoPainter (CP) and FineSTRUCTURE (FS) we reveal a highly geographically clustered genetic structure in Finland and report its connections to the settlement history as well as to the current dialectal regions of the Finnish language. The main genetic division within Finland shows striking concordance with the 1323 borderline of the treaty of Nöteborg. In general, we detect genetic substructure throughout the country, which reflects stronger regional genetic differences in Finland compared to, for example, the UK, which in a similar analysis was dominated by a single unstructured population. We expect that similar population genetic reference data sets will become available for many more populations in the near future with important applications, for example, in forensic genetics and in genetic association studies. With this in mind, we report those extensions of the CP + FS approach that we found most useful in our analyses of the Finnish data.

Methods for estimating fine-scale genetic structure are becoming increasingly important for genetics research. First, an optimal design of rare variant association studies requires knowledge of detailed genetic structure because rare variants are often population specific and geographically clustered (The 1000 Genomes Project Consortium *et al.* 2015). Second, as the well-established methods to control for genetic ancestry in common variant association studies do not necessarily work well for rare variants (Mathieson and McVean 2012), we need new approaches to appropriately adjust the ongoing sequencing studies for fine-scale population structure. Third, fine-scale genetic structure can refine relationships between closely related populations and reveal recent history, including population movements over the last centuries (Genome of the Netherlands Consortium 2014; Karakachoff *et al.* 2015; Leslie *et al.* 2015; Athanasiadis *et al.* 2016). Novel methods can even provide useful estimates of an individual’s recent past within countries considered to be genetically homogeneous, such as the UK (Leslie *et al.* 2015). We expect that this opportunity will have an important role in the near future in engaging the general public to participate in large biobank collections or community efforts for genetics research, such as DNA.Land or Genes for Good (Check Hayden 2015). Finally, an accurate estimate of biogeographic ancestry of a DNA sample is important in forensic genetics (Kayser and de Knijff 2011).

Recently, the estimation of fine-scale genetic structure has improved due to increased sample sizes and advancements in statistical modeling (Novembre and Peter 2016). In particular, utilization of haplotype information captures more detailed genetic ancestry than standard methods based on independent variants (Gattepaille and Jakobsson 2011; Lawson *et al.* 2012; Duforet-Frebourg *et al.* 2015). A promising approach to exploit haplotype information combines software packages ChromoPainter (CP) and FineSTRUCTURE (FS) (Lawson *et al.* 2012). CP summarizes the genetic similarity of the samples in a coancestry matrix that, for each individual, contains estimates of the proportion of his/her genome that is the closest with each of the other individuals in the sample. FS then clusters the individuals into populations via a nonparametric Bayesian model based on the coancestry matrix from CP. Leslie *et al.* (2015) recently applied CP + FS to the Peoples of the British Isles project data (Winney *et al.* 2012) and reported striking concordance between genetic clusters and geography. For example, they genetically differentiated the neighboring counties of Cornwall and Devon in southwest England. We expect that, in the near future, the landmark work of Leslie *et al.* will motivate fine-scale analyses in many other populations, as well as new applications of CP + FS to individual-level fine-scale ancestry estimation within countries and regions that have so far been considered genetically too homogeneous for such analyses. Extending the interpretability of the output from CP + FS and evaluating how robust CP + FS is to parameters such as the sample size and sampling density of individuals is therefore timely.

In this work, we apply CP + FS to a Finnish population sample both of whose parents were born within 80 km of each other. Finland with its relatively small founder population and strong genetic isolation (Norio 2003b; Salmela 2012) has become one of the most widely utilized populations in genetic studies of diseases and traits (Peltonen *et al.* 1999; Sabatti *et al.* 2009; Lim *et al.* 2014). Our goal was to characterize the fine-scale genetic population structure within Finland before migrations that have occurred from 1950s onwards both to serve as a reference data set for ongoing and future genetic association studies as well as to reveal relationships between genetics, known historical events, and the dialectal groups of Finland.

First, we refine our knowledge about the relatively strong genetic difference between western (W) and eastern (E) parts of the country (Lappalainen *et al.* 2006; Jakkula *et al.* 2008; Salmela *et al.* 2008; Neuvonen *et al.* 2015). Previous genetic analyses have studied this difference by collecting individuals from the opposite sides of the country and observing that their genetic differentiation is large compared to differentiation between some European countries, such as the UK and Germany (Salmela *et al.* 2008). By utilizing autosomal haplotype information from individuals that uniformly cover the main part of Finland we locate an explicit genetic borderline between W and E Finland, and we introduce a Gaussian mixture model to assess its uncertainty. We find strong similarities between the genetic borderline and both the treaty of Nöteborg from 1323 and the settlement history of Finland (Figure 1).

**Figure 1:**
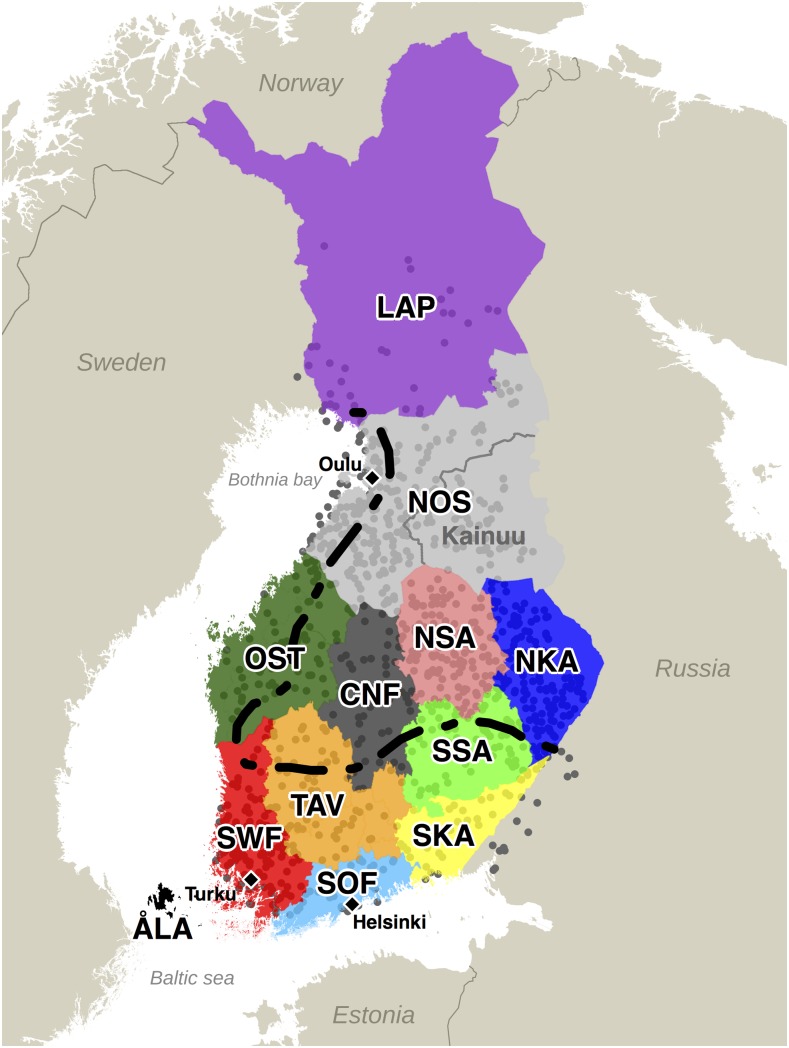
Locations of 1042 samples and the 12 Finnish provinces (1996 definition). Each sample is at the mean of parents’ coordinates. LAP: Lapland, NOS: Northern Ostrobothnia, OST: Ostrobothnia, CNF: Central Finland, NSA: Northern Savonia, SSA: Southern Savonia, NKA: Northern Karelia, SKA: Southern Karelia, TAV: Tavastia, SWF: Southwestern Finland, SOF: Southern Finland. Kainuu is a subregion of NOS. The dashed line divides Finland into an early-settlement area (south and west of the line) and a late-settlement area (north and east of the line) (Jutikkala 1933). Cities of Helsinki, Turku, and Oulu are marked with black diamonds.

Second, we catalog the Finnish population structure at a finer scale, identifying nearly 20 geographically clustered populations that overlap minimally with each other in general and cover approximately similar surface areas of the country. We find striking concordance between many of the genetic populations and the dialectal regions of the Finnish language. We validate the robustness of the fine-scale structure to characteristics of the data such as the sample size and sampling density of the individuals and study the relationships of these populations by comparing two approaches for building a hierarchical tree.

In *Results*, we report the fine-scale analysis of the Finnish population structure and assess its robustness. Connections to the earlier work on the Finnish population structure are given in *Discussion*. We have made our results available through a website (see *Data availability*).

## Materials and Methods

### Samples

The FINRISK Study is a representative, cross-sectional survey of the Finnish working age population (age range 25–74) that, since 1972, has collected a random sample of 6000–8000 individuals every 5 yr to study risk factors of chronic diseases in Finland. Our data were from the FINRISK Study survey of 1997 (Vartiainen 1998) and included genotype data of 4191 individuals born between 1922 and 1972 and their parent’s birth municipalities. The study protocol of the FINRISK Study 1997 was approved by the Ethics Committee of the National Public Health Institute (decision number 38/96). All participants gave written informed consent. To obtain a geographically precisely defined sample, we took forward only those 2376 individuals both of whose parents were born within 80 km from each other and who passed the quality control criteria defined below. The distance between parents was calculated using the great-circle distance and the coordinates of the city centers of the birth municipalities of the parents. The coordinates of the individuals were calculated as an average of their parents’ coordinates. As the youngest individuals in our sample were born in 1972, it follows that almost all parents of our samples were born before 1950. Hence, our data reflect the population structure of Finland before internal migration events that have taken place since around 1950.

### Genotypes

The genotyping was performed with Illumina HumanCoreExome-12 BeadChip at the Wellcome Trust Sanger Institute, Hinxton, United Kingdom. Genotyping success was first checked at the Sanger Institute after which we performed additional quality control steps by excluding SNPs with minor allele frequency (MAF) below 5%, Hardy–Weinberg equilibrium *P*-value below 10^−6^, or call rate below 99.9%. This resulted in 238,438 SNPs. For the CP analysis with rare variants we ignored the MAF filter and included all SNPs with minor allele count above 1 resulting in 303,221 SNPs. All MAFs, HWE values, and call rates were calculated using PLINK version 1.07 (Purcell 2007).

### Sample quality control

We excluded the individuals that stood out from the other samples with average heterozygosity |F| > 0.025 or variant missingness rate >0.003. We also excluded individuals on two genotyping plates with poor quality. We calculated the relatedness for each pair of individuals using both PLINK 1.07 (Purcell 2007) and GCTA 1.24.4 (Yang *et al.* 2011) and excluded one individual from each pair for which either one of the relatedness values exceeded 0.05.

### Uniform sample selection

As the genotyping of the individuals from the FINRISK Study survey of 1997 upweighted Eastern Finland in its sampling, about half of the full data set of 2376 individuals were located in the provinces of Northern Karelia and Northern Savonia (Table 1). To study how the uneven sampling density or variation in the total sample size affected the FS results, we constructed three more uniformly distributed subsets of the data. We first placed a grid of 25 km on a map of Finland and sampled at maximum one, two or five randomly chosen individuals from each square. This sampling resulted in data sets that consisted of 328, 580, or 1042 individuals, respectively. We considered the data set with 1042 individuals as our main data set.

**Table 1:** Sample sizes

Province	Full Data Set	Main Data Set
Lapland (LAP)	38	38
Northern Ostrobothnia (NOS)	522	263
Kainuu*^a^*	140	57
Northern Savonia (NSA)	592	139
Northern Karelia (NKA)	587	139
Central Finland (CNF)	45	45
Southern Savonia (SSA)	90	69
Southern Karelia (SKA)*^b^*	49	47
Ostrobothnia (OST)	85	84
Tavastia (TAV)	75	71
Southwestern Finland (SWF)	226	109
Southern Finland (SOF)	67	38
Åland (ÅLA)	0	0
Total	2376	1042

a Kainuu samples are included in NOS samples.

b Includes samples outside the southeastern border.

### ChromoPainter and FineSTRUCTURE analyses

The genotype data (after QC) were phased jointly for all individuals with SHAPEIT2 (Delaneau *et al.* 2013) using default options and the effective population size 11,418 (European average). A recombination map was obtained for the genome build 37 (http://www.shapeit.fr/files/genetic_map_b37.tar.gz, downloaded 25.6.2014).

Population structure analyses were performed similarly for all four data sets using ChromoPainter 0.0.4 and FineSTRUCTURE 0.0.4 (FS) programs (Lawson *et al.* 2012). Phased genotype files were converted into CP format and global switch and emission rates were estimated using CP’s expectation-maximization algorithm (10 iterations) on chromosomes 1, 9, 15, and 22 using averages over 24 individuals. We also verified these estimates using an almost 10-fold larger sample of 238 individuals, and the estimates did not notably change. [Recently, Leslie *et al.* (2015) reported that a 10-fold difference in the switch rate does not have a big impact on the results.] CP was then run using the estimated global parameters and the HapMap build 37 recombination map converted into the CP format.

Population assignment was performed with FS that reads in CP’s chunkcounts output and assigns individuals into genetically (relatively) homogenous groups using a nonparametric Bayesian mixture model implemented through a Markov Chain Monte Carlo (MCMC) algorithm [more details in Lawson *et al.* (2012) and Leslie *et al.* (2015)]. FS was run with the default options of 1,000,000 burn-in iterations, 1,000,000 MCMC iterations from which every 10,000th iteration was recorded. FS-tree was built using 1,000,000 tree comparisons and 100,000 additional hill climbing moves. We ran all four data sets without a predefined number of populations and we also ran the data set with 1042 individuals by specifically asking for two populations (Figure 3).

After the FS analysis, we performed an additional step to improve the population assignment by maximizing the overall posterior probability. This was done as in Leslie *et al.* (2015).

### Estimating population uncertainty

To assess the probability of each individual belonging to either W or E population, we applied a semisupervised Gaussian mixture model (GMM) on CP’s coancestry matrix. For each individual *i*, we calculated a logarithm of ratio of average E *vs.* W chunks as

$$x_{i}=\log\frac{\frac{1}{n_{e}}\sum_{e=1}^{n_{e}}c_{ie}}{\frac{1}{n_{w}}\sum_{w=1}^{n_{w}}c_{iw}},$$

where *n_e_* is the number of eastern and *n_w_* the number of western reference individuals and *c_ie_* and *c_iw_* are the number of chunks in coancestry matrix that individual *i* copied from an eastern individual *e* or a W individual *w*. The eastern reference individuals included those individuals from the province of Northern Karelia (NKA) who belonged to the E population in FS results with two populations (Figure 3A) and, accordingly, the W reference individuals included those in the province of Southwestern Finland (SWF) who belonged to the W population. The provinces of NKA and SWF were selected as they represent the far E and far W corners of Finland. Then, we fitted a supervised GMM on the *x_i_* values using an EM-algorithm (publicly available, see *URLs*).

### Principal component analysis (PCA)

To compare the chromosome painting method with standard methods that use only unlinked markers, we performed PCA with SmartPCA of EIGENSOFT package (Patterson *et al.* 2006). We ran SmartPCA on 61,598 SNPs that were pruned to have *r*^2^ < 0.2 within 1 cM windows and excluded the long-range linkage disequilibrium (LD) regions according to Price *et al.* (2008). We performed a PCA on CP’s coancestry matrix as in Lawson *et al.* (2012), that is, by adding the column sums to the diagonal, by subtracting the column means from the elements, and by making the matrix symmetric by multiplying it with its transpose.

**Figure 2:**
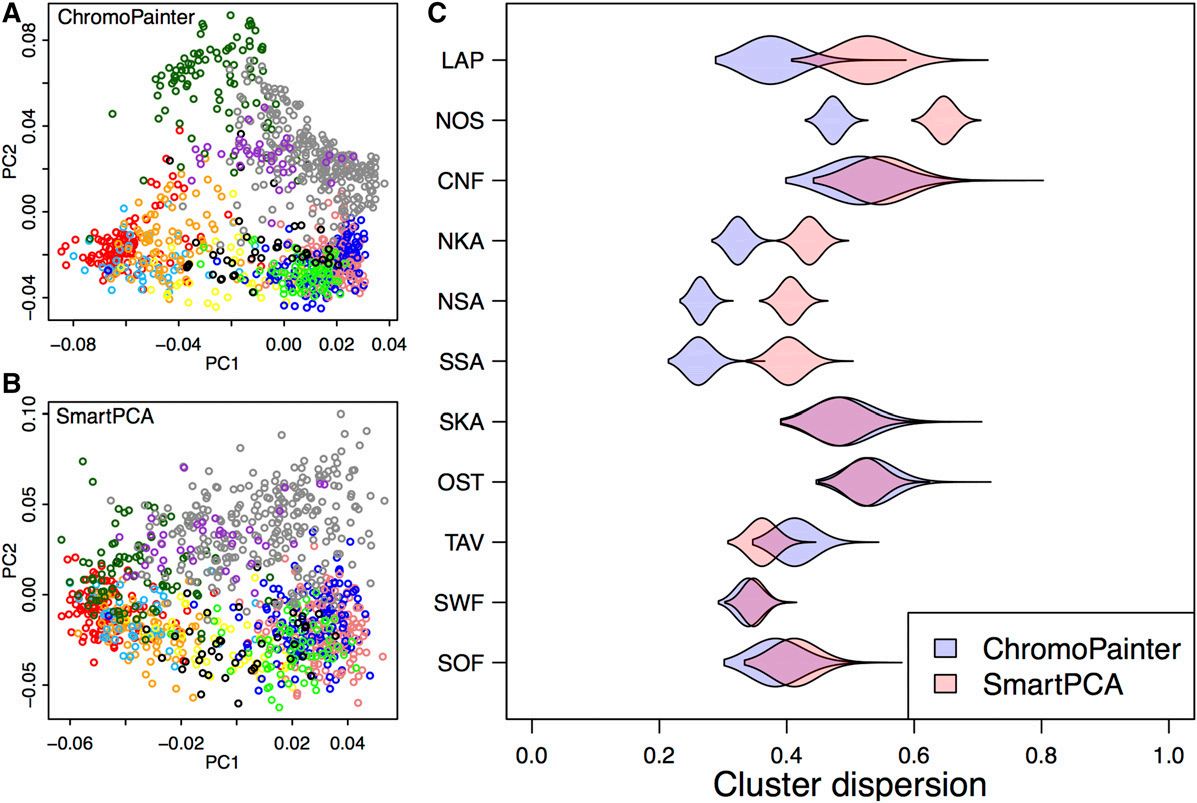
(A and B) The first and second principal components of genetic structure given by ChromoPainter (A) and SmartPCA (B) with individuals colored according to provinces of Figure 1. (C) For each province, the violin plots show how dispersed, as measured by the sample variance, the individuals from that province are in A and B compared to a random set of similar size (*Materials and Methods*).

The comparison (Figure 2C) was performed by calculating, for each group, the average squared distance from the group mean, *i.e.*, the empirical variance on a plane defined by the first and the second principal components. This variance was then scaled to correspond the variance of a similar sized random sample of individuals from the same two-dimensional principal component (PC) plot, which made it possible to compare the two PCA plots. We performed 100,000 random samplings for each group and show their distribution using violin plots in Figure 2C. The groups were defined by the Finnish provinces shown in Figure 1.

To study the effect of including rare variants in CP analyses, we did a similar comparison between our main data set and the data set that also included the rare variants (Supplemental Material, Figure S1 in File S1).

### The border of the treaty of Nöteborg

The exact border of the treaty of Nöteborg is well defined only from Vyborg castle through Äyräpää and Jääski to the southeastern parts of present-day Finland (Katajala 2012). Several studies have speculated how the border continues toward Western Finland ending somewhere between Kalajoki and Pattijoki (Juhola 2011). Thus, we decided to draw our approximate border from Jääski (28.92 N, 61.04 E) to Pyhäjoki (24.26 N, 64.46 E), which is half way between Kalajoki and Pattijoki and which has also been suggested to be a possible western end point of the border (Vilkuna 1960; Julku 1987).

### Total variation distance (TVD) and TVD-tree

To calculate a genetic distance between populations that takes into account the haplotype information but does not depend on the sample sizes of the populations, we calculated TVD as described in Leslie *et al.* (2015). First we fixed K, the number of reference populations (*e.g.*, *K* = 17 in TVD-tree of Figure 4B), and then we defined, for each individual, a copying vector of length K whose element k tells which proportion of the individual’s genome is copied from population k. For a population, the copying vector is an average over the individuals in that population. TVD summarizes the differences between two populations, *a* and *b*, by comparing copying vectors as

$$TVD_{a,b}=0.5*\sum_{i=1}^{K}\left|\left(a_{i}-b_{i}\right)\right|,$$

where *a_i_* (*b_i_*) is population *a*’s (*b*’s) copying proportion from population *i*. We verified that the choice of the number of reference populations has only a minor effect on TVD (Figure S10 in File S1). We built TVD-tree in K-1 steps by, at each step, calculating TVD for each pair of the current populations and merging those two populations whose TVD was the smallest. In particular, TVD was at each step computed with respect to the K dimensional copying vectors even though the number of current populations decreased by one at each step. We visualized the tree by scaling the branch lengths proportional to the TVD value of the corresponding pair of populations. The code is publicly available (see *URLs*).

### Pairwise F_ST_

**Figure 3:**
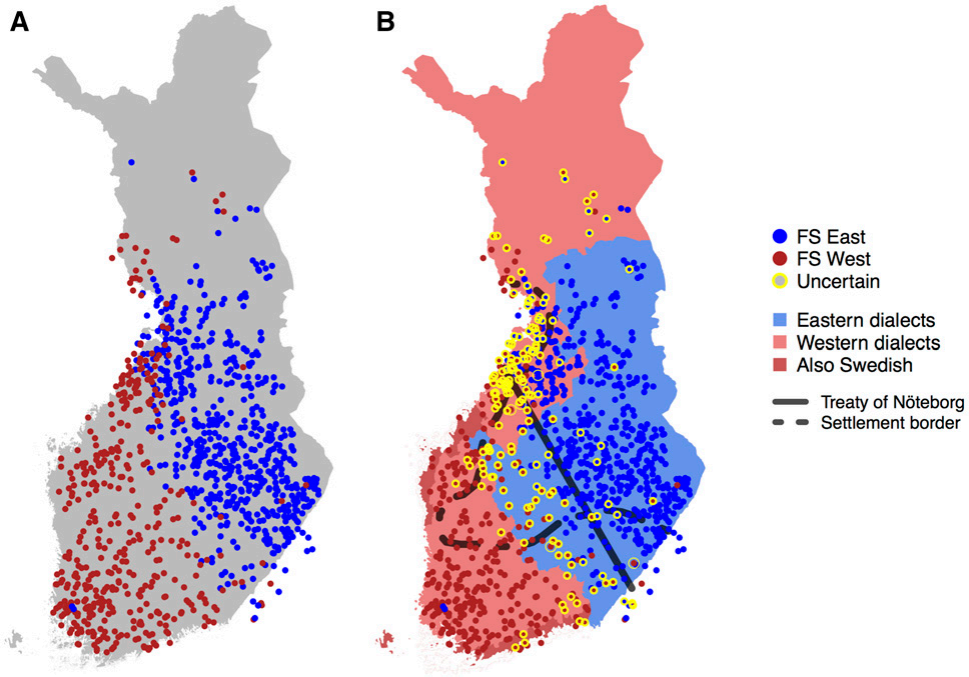
(A) FineSTRUCTURE results with two populations that we labeled west (W) and east (E). (B) Results from A refined by marking with yellow circles the individuals whose assignment is uncertain (<80% assignment probability to both populations). Also shown are the approximate 1323 borderline of the treaty of Nöteborg, the early *vs.* late-settlement border from Figure 1, and the regions of E and W dialects of the Finnish language, including partly Swedish-speaking coastal regions.

**Figure 4:**
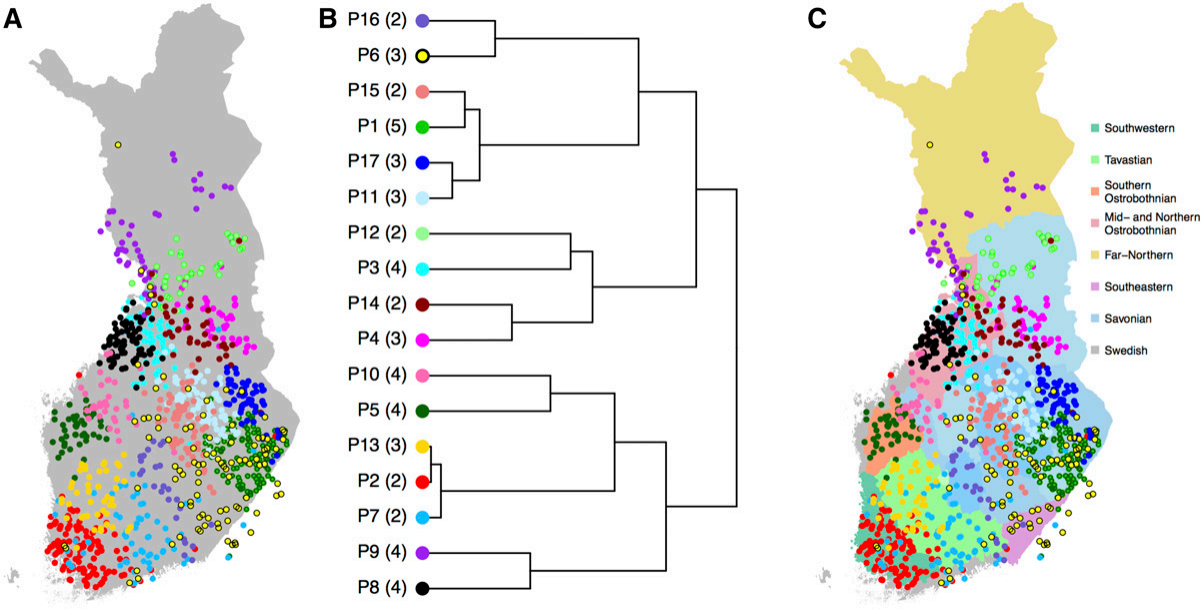
(A) Fine-scale population structure with 17 populations, (B) their relationships according to TVD-tree, and (C) their overlap with the seven main dialectal regions of the Finnish language with eight Savonian subdialects marked with different shades of blue. Numbers in parentheses in B show into how many subpopulations these 17 populations split in the complete tree of 52 populations (Figure S3 in File S1).

Pairwise F_ST_ measures genetic differentiation by comparing allele frequencies between two populations. We estimated pairwise F_ST_ values between the main W and E division (Figure 3A) and between the 17 fine-scale populations (Figure 4A) using the 61,598 SNPs from the PCA analysis and Hudson’s F_ST_ estimate (Bhatia *et al.* 2013) implemented in the EIGENSOFT package (Patterson *et al.* 2006).

### The names of the Finnish provinces

We have used geographically motivated, simple English names for the 12 Finnish provinces (1996 definition). The official Finnish names of these provinces are LAP: Lapin lääni, NOS: Oulun lääni, OST: Vaasan lääni, CNF: Keski-Suomen lääni, NSA: Kuopion lääni, SSA: Mikkelin lääni, NKA: Pohjois-Karjalan lääni, SKA: Kymen lääni, TAV: Hämeen lääni, SWF: Turun ja Porin lääni, SOF: Uudenmaan lääni, ÅLA: Ahvenanmaan lääni.

### URLs

Scripts for estimating population assignment probabilities and TVD are available at http://www.helsinki.fi/∼sinikerm/; PLINK, http://pngu.mgh.harvard.edu/∼purcell/plink/; GCTA, http://cnsgenomics.com/software/gcta/; SHAPEIT2, https://mathgen.stats.ox.ac.uk/genetics_software/shapeit/shapeit.html; ChromoPainter and FineSTRUCTURE, http://paintmychromosomes.com/; EIGENSOFT, https://www.hsph.harvard.edu/alkes-price/software/.

### Data availability

The genotype data used in this study are available through the National Institute for Health and Welfare Biobank https://thl-biobank.elixir-finland.org/. Results for fine-scale structure at different levels are available at https://www.fimm.fi/en/research/projects/finnpopgen. File S1 includes Figures S1–S10 and File S2 includes Tables S1 and S2.

## Results

We characterized the genetic population structure in Finland using data from the FINRISK Study survey of 1997 (Vartiainen 1998). We first identified a set of 2376 individuals both of whose parents were born within 80 km from each other and that did not contain close relatives (*Materials and Methods*). This sample covered 10 out of 12 provinces of Finland well (1996 definition of provinces) with the exceptions of Lapland (only a few individuals) and Åland (no individuals at all). There were large differences in the sampling density also across the other provinces and therefore our main analysis used a subset of 1042 individuals with a more uniform spatial distribution (Figure 1 and Table 1).

All our samples were genotyped using Illumina HumanCoreExome-12 BeadChip. Our main analysis used 238,438 directly genotyped SNPs with MAF > 5% that passed the quality control metrics (*Materials and Methods*).

### Chromosome painting

Generating a haplotype-based coancestry matrix using CP requires considerably more computational resources than calculation of the empirical correlation matrix across a set of independent variants. Therefore, we started by evaluating whether the higher computational cost of CP is compensated by CP capturing more information than the standard relatedness matrix based on independent SNPs. Figure 2 shows the first two PCs of both approaches [panel A for CP and panel B for SmartPCA (Patterson *et al.* 2006) that uses the empirical correlation matrix] and quantifies how dispersed (panel C; *Materials and Methods*) the individuals from the 11 provinces are in these two PC plots compared to a random set of individuals. CP clearly clusters the individuals from five provinces (LAP, NOS, NKA, NSA, SSA) tighter than SmartPCA whereas the opposite is true only for the province of TAV. In the remaining five provinces, we see little difference between the two methods. These results illustrate CP’s overall tendency to cluster individuals who live geographically closer more tightly together than SmartPCA, especially in the northern and eastern parts of the country, which we expect to be the most genetically isolated due to their later permanent inhabitation by a relatively small set of individuals starting from the 1500s (Figure 1 and *Discussion*).

We also assessed whether an addition of 64,783 low-frequency and rare variants (MAF < 5%) available on the genotyping chip affected the CP results but did not observe any noticeable difference compared to the common variant analysis (Figure S1 in File S1). This indicates that in our data, the high-quality common variants sufficiently capture the haplotype structure compared to all available variants.

These results motivated us to then run FS on the CP output of the common variant analysis to reveal fine-scale population structure in Finland.

### Division between Western and Eastern Finland

To establish the high-level genetic structure in Finland, we applied FS to the output of CP by allowing exactly two populations. As expected, the main genetic division was between W and E parts of the country (Figure 3A). The pairwise F_ST_ (Patterson *et al.* 2006) between these two populations was 0.002 (SE = 2 × 10^−5^). The clustering model of FS did not report almost any uncertainty for this binary population assignment (Figure S2 in File S1). To reveal more detailed differences between individuals in the proportions of genome related to the two populations, we used a GMM to assess how certain each individual was to belong to the W or E population based on the CP coancestry matrix and FS output (*Materials and Methods*). In Figure 3B we have marked those individuals who did not belong to either W or E population with over 80% probability. These individuals highlight a genetic border between W and E from the southeastern corner of Finland to the coast of Central Ostrobothnia leaving Southwestern Lapland also closer to the W population.

Next, we compared this genetic border to historical records and dialectal patterns, both showing features of W and E differentiation. The first and the most densely inhabited regions concentrated on Southern Finland and the coastal regions up to the Bothnia bay dividing Finland into the southwestern early-settlement region (ESR) and the northeastern late-settlement region (LSR), which became permanently inhabited from the 1500s (Figure 3B) (Jutikkala 1933). While in general ESR is covered by the W population and LSR is covered by the E population, we point out two exceptions: the ESR provinces of SSA and SKA (Figure 1) are mainly covered by the E population whereas the LSR to the west of CNF is covered by the W population. We discuss these observations together with Southwestern Lapland’s close relation to the W population in *Discussion*.

The first official border within modern day Finland was ratified in the treaty of Nöteborg in 1323 (fin. Pähkinäsaaren rauha), and it joined the southwestern part of Finland to the Kingdom of Sweden and Eastern Finland to Novgorod (a historical state located in modern day Russia). In Figure 3B we have approximated the border by a line between Jääski near the southeastern border of Finland and Pyhäjoki (see *Materials and Methods*) on the coast of Ostrobothnia (Katajala 2012). The genetic division between the W and E populations follows this medieval border line strikingly accurately, leaving more individuals with uncertain assignment on the southern side of the border (Figure 3B).

The primary dialectal division of the Finnish language is into E and W dialects (http://www.kotus.fi/kielitieto/murteet/suomen_murteet, 2015 and Itkonen 1989), shows an overall concordance with the genetic division, with an exception on the northern side of the 1323 border near Oulu where the W dialects overlap with the E population (Figure 3B).

### Fine structure

When FS was run without a preassigned number of populations, it divided our sample of 1042 individuals into 52 populations (Figure S3 in File S1). As an example of fine-scale genetic structure in Finland, Figure 4A shows 17 populations from the default hierarchical tree of FS on the map of Finland. We chose this level of the tree because it already reveals detailed population structure without introducing very small populations (*i.e.*, < 25 individuals) and because we have verified its robustness to sample size included in the analysis by a comparison with another subset of the data (Figure S4 in File S1). Figure 4A shows that overall the populations are geographically clustered, overlap little, and are distributed evenly across Finland. The only exception from tight clustering is P6, which exhibits diffuse clustering along the E–W borderline, as identified in Figure 3, and includes individuals around the large southern cities of Helsinki and Turku as well as around the northern city of Oulu, 540 km north of Helsinki (see Figure 1 for cities on map and *Discussion* for more information about this population). The pairwise F_ST_ values corresponding to these 17 populations (Table S1 and Table S2 in File S2) show that overall P6 has relatively small F_ST_ values with all other populations indicating approximately equal relatedness to both E and W populations.

To visualize the hierarchical structure of these populations, we compared two agglomerative clustering algorithms to build a hierarchical tree for the populations. FS provides an algorithm (here: FS-tree) that at each level of the tree building merges the two populations resulting in the highest posterior probability among all possible merges. Lawson *et al.* (2012) reported that although FS-tree has performed well in practice, it might depend significantly on the sample sizes of the populations. We therefore compared FS-tree to another tree-building algorithm based on TVD between populations (TVD-tree, *Materials and Methods*) that does not depend on the sizes of the populations. In Figure 4B we show TVD-tree for these 17 populations because, in our data, TVD-tree produced more consistent results across different sample sizes than FS-tree (see *Sample size and sample density*). Figure 4B shows that after the E–W split, the next split in the east is between Kainuu and Southeastern Finland, and in the west is between Northern and Southwestern Finland. When we follow the more detailed tree to its 52 leaves (Figure S3 in File S1), these four regions (Kainuu, SE Finland, N Finland, and SW Finland) split into 11 (178 individuals), 18 (427), 8 (123), and 15 (314) populations, respectively. Hence, we observe fine-scale population structure across the whole of Finland, which is in contrast to an FS analysis of the UK of the late 1800s (Leslie *et al.* 2015) where a large unstructured population covered a major part of the country. However, we note that in TVD-tree, the southwest corner of Finland, which has been permanently inhabited the longest (Jutikkala 1933), is the last region to split into smaller parts both in Figure 4B and in the TVD-tree of all 52 populations (Figure S3 in File S1).

In addition to the primary E–W dialectal division (Figure 3B), the Finnish dialects are further divided into seven main dialects and their subdialects. Figure 4C overlays the main dialects with the genetic populations and shows that, on many occasions, the genetic populations closely follow the dialectal borders. In Western Finland the regions of Southwestern, Tavastian, Southern Ostrobothnian, Mid and Northern Ostrobothnian, and Far-Northern dialects show primarily one or two populations located exclusively at each region. For example, P5 is strictly located at the Southern Ostrobothnian dialectal region and P2 at the Southwestern dialectal region. Only near the city of Oulu do we see a mixture of individuals from several populations whose primary location is outside this dialectal region. In Eastern Finland, the Savonian dialectal region covers several genetic populations but even there the concordance between genetics and dialects can be detected when compared to subdialectal regions (Figure 4C). Figure 4C also reveals an interesting detail about the Savonian dialect spoken in Ostrobothnia in Western Finland. Indeed, we observe a genetic population (P10) that clusters in this region, but genetically this population is closer to other populations in Western Finland than to the populations in Eastern Finland. The southeastern dialectal region lacks a unique genetic population of its own since P6, which covers this region, is also widely spread out geographically to the Savonian dialectal region.

### Sample size and sample density

To study how sample size and sampling density affect the FS results, we compared our main data set of 1042 individuals to two additional subsets of our data with 328 and 580 individuals as well as to the full data set of 2376 individuals. Individuals in the three subsets were geographically evenly distributed (*Materials and Methods*) while the full data set contained considerably more individuals in the regions of NSA and NKA (Figure 1 and Table 1). We analyzed all data sets with the same pipeline and observed that the total number of populations detected by FS increased approximately linearly with the number of individuals (14, 22, 52, and 170 populations for 328, 580, 1042, and 2376 individuals, respectively). We next investigated how this correlation between the number of populations and the sample size affected the main properties of the population structure identified from each data set.

**Figure 5:**
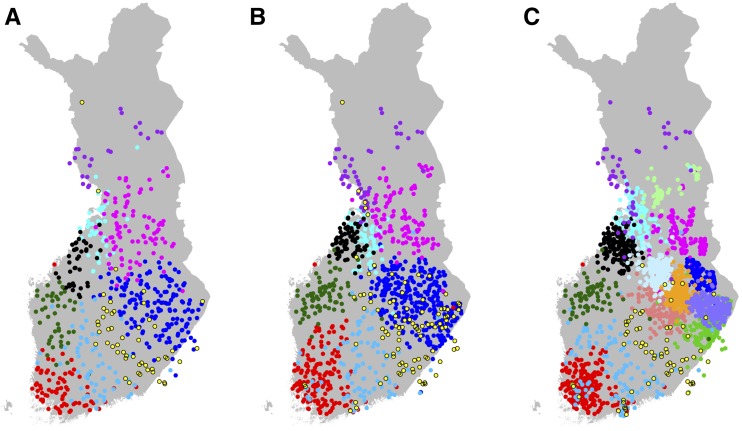
FS results with varying sample size and sample density. (A) Data set of 580 individuals at FS-tree level 9, (B) data set of 1042 individuals at FS-tree level 9, and (C) data set of 2376 individuals at FS-tree level 15.

The visual comparison of the data sets at different levels showed that we can recognize core populations that exist in every data set. In Figure 5, these populations cluster near Oulu (cyan), into LAP (purple), Kainuu (magenta), NSA and NKA (blue), SSA and SKA (yellow), Central Ostrobothnia (black), OST (dark green), TAV (sky blue), and Southern Finland (red). (Results of sample size of 328 are shown in Figure S5 in File S1.) In the more uniformly distributed data sets (Figure 5, A and B and Figure S5 in File S1), these are the nine populations first to split in FS-tree but with the data set of 2376 individuals (Figure 5C) we recognize all nine populations only when 15 populations are observed. Figure 5C shows that the additional six populations of the data set with 2376 samples are all located in Eastern Finland where the sampling was densest. This observation suggests unsurprisingly that FS identifies more populations where the sample is denser due to increased statistical power for population detection in those regions, but also that these populations can occur at relatively close to the root of FS-tree compared to subsets of data with a more uniform sampling strategy. This raises concerns about whether the splitting order given by FS-tree primarily reflects the genetic differences, or whether it is also significantly affected by varying sample sizes between different populations. While a bifurcating tree is only an approximation to the complex relationships between the populations, we can at least test how stable the structure of the tree is across varying sample sizes and sampling densities using either FS-tree or TVD-tree.

When we compared the FS-trees and TVD-trees (Figure S5–S8 in File S1) we observed that the first split divides all the data sets into E and W but in the two largest data sets (1042 and 2376 individuals), the division made by FS-tree assigned essentially all individuals above the treaty of Nöteborg to the E population, which did not match with the main genetic E–W split identified earlier by explicitly modeling two populations (Figure 3). In contrast, in all four TVD-trees as well as in the two remaining FS-trees (328, 580) the E–W split is consistent with Figure 3. We emphasize the TVD-tree rather than an FS-tree in Figure 4B because TVD-trees were more consistent than FS-trees both across the sample sizes and in comparison with the explicit analysis of the main genetic split within Finland.

## Discussion

We anticipate that fine-scale genetic structure estimation will become an essential part of future rare variant association studies and individual-level ancestry estimation across the globe. We assessed performance and robustness of the haplotype-based methods ChromoPainter (CP) and FineSTRUCTURE (FS) in revealing fine-scale genetic structure in Finland. First, we defined geographically the main genetic division between Eastern and Western Finland using precisely located samples based on parents’ birthplaces. Second, we characterized the fine-scale genetic population structure present before the 1950s. Our results serve as a population genetic reference for future design and interpretation of genetic association studies and individual-level ancestry estimation in Finland. We validated CP + FS results by comparing them to a standard PCA, incorporating a more sensitive uncertainty measure, comparing different ways of building the hierarchical structure among the populations, and studying the effect of sample size and sampling density on our results. In general, we found CP + FS to be a useful and robust method for fine-scale population structure analysis with a few caveats that will be important to take into account in future applications in other populations. We note that our analyses do not explicitly model migrations and admixture events and future studies with complementary approaches and complementary data from the neighboring countries of Finland are required to study these topics.

Finland has, for the last 10,000 yr, been a border region between W populations of Scandinavia, southern populations of the Baltics, and E populations of European Russia as summarized by Salmela (2012). These long-term influences may have contributed to the main genetic division within Finland separating W and E parts of the country (Figure 3A). Another contributing factor to this primary split is likely to be the relatively small population size and isolated nature of many parts of the late-settlement areas concentrated in Eastern and Northern Finland (Figure 1) that, according to historical records, were only sparsely inhabited, if at all, until the middle of the 16th century when people, mainly from Southern Savonia (SSA), gradually extended their practice of agriculture and more stable habitation to these areas (Jutikkala 1933).

Consistent with Southern Savonian settlers inhabiting Eastern and Northern Finland, we indeed observe that SSA, and its neighboring province SKA, are the only areas of the early-settlement region that are primarily covered by the E population that extends from SSA to the LSR (Figure 1 and Figure 3B). On the other hand, the southwest corner of the LSR is genetically part of the W population rather than the E population. A possible historical explanation for this is that these areas were old hunting grounds of Tavastians and therefore may have attracted Tavastian settlers in addition to Savonians (p. 99, Jutikkala 1933). Also, later contacts between this region and the neighboring coastal areas of the early-settlement region of Ostrobothnia may well have contributed to the major influences from the W population that we observe in this region today.

Our analysis established that the 1323 borderline of the treaty of Nöteborg is a very accurate description of the main genetic split within Finland (Figure 3B). This may support a role for the 16th century Swedish authorities in guiding the Savonian settlers to inhabit land particularly to the east of the 1323 border (p. 98, Jutikkala 1933). However, it seems unlikely that the 1323 border itself would have been a physical cause of the genetic population structure as the border was of a more administrative nature and did not restrict the movements of common people (Katajala 2012; Korpela 2002).

An interesting detail of the split between the W and E populations is a group of individuals in Torne Valley (fin. Tornionlaakso) in Southwestern Lapland who are assigned to the W population even though geographically they are separated from the rest of the W individuals by E individuals near Oulu (Figure 3A). While it is possible that the early-settlement region on the west coast had also extended to Torne Valley (p. 67–68, Jutikkala 1933), establishing a genetic connection all the way to Southern Finland, it is also possible that the close ties between the Finnish side of Torne Valley and Sweden across the Torne River have resulted in a genetic admixture whose Swedish component clustered these individuals with Western Finland regardless of how their Finnish component would have clustered them.

Two previous studies about population structure in Finland using genome-wide autosomal variants have either not attempted to define populations based on genetic data (Jakkula *et al.* 2008) or have done so by applying the STRUCTURE algorithm (Pritchard *et al.* 2000) to an independent set of 6369 variants (Salmela *et al.* 2008), which did not reveal the fine-scale genetic structure within Finland. Our haplotype-based population assignment using a data set that evenly covers a major part of Finland therefore provides unprecedented information on the fine-scale genetic structure of the Finnish population. In general, the fine-scale structure that we detected is highly geographically clustered with little overlap between the populations (Figure 4 and Figure 5 and Figures S4–S8 in File S1). There are no large differences in the area covered or number of individuals included in each population (with the exception of P6 in Figure 4). This is likely a consequence of a relatively small population size and isolation by distance throughout the country. It is instructive to contrast this pattern to an FS analysis in the UK that focused on the population structure of the late 1800s (Leslie *et al.* 2015). In this analysis, a single population covered central and southern England and included almost half of all the individuals, even at the finest level where the sample was already split into 53 populations (Leslie *et al.* 2015). The strong genetic clustering within Finland is in line with the pocketed distribution of multiple severe diseases of the Finnish Disease Heritage (Peltonen *et al.* 1999; Norio 2003a) and suggests that Finland also holds great promise for future studies on less severe and more prevalent diseases for which genetic variants with large effects have been more difficult to identify.

In our analysis, the exceptionally dispersed P6 dominating the Southern Karelia region also spread out widely across Savonia, near the large southern cities of Helsinki and Turku as well as along the genetic East–West borderline of Figure 3 all the way to Oulu and even further north (Figure 4A). As a hint that this dispersal might be due to recent events, we noticed that individuals of P6 were clearly younger than the rest of our samples (median birth years 1957 and 1950, respectively; Mann-Whitney *P*-value 10^−5^). When we further split P6 into its three subpopulations (Figure S9 in File S1), we noticed that one of these remained stable across the birth years of our samples (orange population in Figure S9 in File S1) while the other two spread out from Karelia to Savonia (green population) and from the city of Vyborg (fin. Viipuri) throughout the country (yellow population) within this timeframe (1922–1972). During and after the Second World War (1939–1945), ∼400,000 Finns from the larger Karelia region southeast of the current borders of Finland were relocated throughout the other parts of Finland. Hence, the youngest third of our sample born after 1957 could, in principle, have such relocated Karelians among their grandparents. However, Figure S9 in File S1 also shows that the dispersal of P6 has started already among individuals born in 1941–1957, and therefore we cannot entirely explain the dispersal by relocations during and after the war (unless some parental birthplaces of these individuals were wrongly reported).

In order to reveal details of the Finnish population structure, we extended CP + FS output in two ways. First, we devised a GMM to estimate uncertainty of population assignment because FS does not report any uncertainty estimate by default. With GMM we can stratify individuals based on the proportion of their genome related to each source population, which information is not available in the FS output. In our analysis, we clustered individuals across Finland based on their genetic makeup with respect to canonical northeastern (Northern Karelia) and southwestern (Southwest Finland) reference samples (Figure 3B). Quantitatively, our uncertainty estimates were clearly larger than those we teased out from the raw MCMC output of FS, although we identified qualitative similarities between the two (compare Figure 3B with Figure S2 in File S1). We note that the two approaches are not estimating the same quantity: Our GMM estimates mixture proportions of an individual originating from each predefined source population, while FS carries out an unsupervised clustering without admixture modeling.

Second, we introduced TVD-tree to complement the default FS-tree for building a hierarchical tree structure for the populations. Traversing an FS-tree from its leaves toward its root, small populations quickly merge to larger ones since small changes to the population assignment cause a relatively small decrease in the model probability. Instead, TVD-tree uses a difference in average ancestries between two populations and hence is less dependent on the sample sizes of the populations. To combine the complementary properties of FS-tree and TVD-tree, we used FS-tree to choose the level of populations (*e.g.*, 17 in Figure 4 and 9 or 15 in Figure 5) but then described the relationships between those populations using TVD-tree. In this way, we avoid including very small populations in the tree, which could be prone to a large sampling variation, while at the same time we make use of TVD-tree, which empirically gave more consistent results across data sets as described in *Results*.

While a discrete population assignment remains an approximation to the underlying genetic relationships between individuals, it provides valuable information for individual-level ancestry estimation and design and analysis of rare variant association studies. We anticipate that our experiences and tools reported here in the context of Finland will be useful for fine-structure analyses of other populations.

## Supplementary Material

Supplemental material is available online at www.g3journal.org/lookup/suppl/doi:10.1534/g3.117.300217/-/DC1.
