# Supplementary material for: Fine-Scale Genetic Structure in Finland
Source: G3 (Bethesda). 2017 Aug 29;7(10):3459–68. doi: 10.1534/g3.117.300217 (PMC5633394; doi:10.1534/g3.117.300217)
Supplement: Supplementary file 2 [file 3459FileS1.docx]

**Supplemental Figures**

**
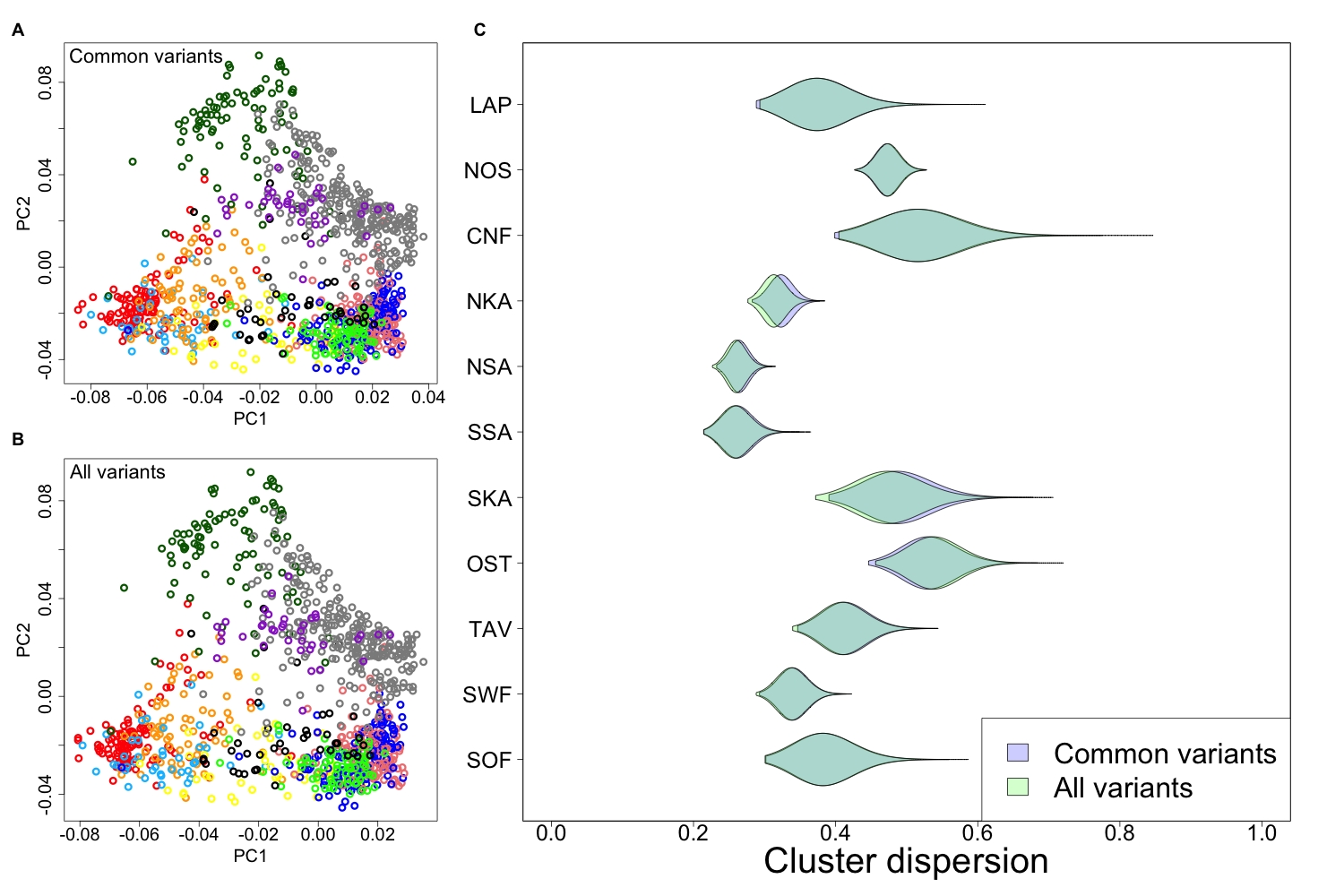
**

**Figure S1 A, B)** The 1^st^ and 2^nd^ principal components (PCs) of genetic structure given by ChromoPainter including the rare variants (panel A) and with only common variants (MAF > 5%) (panel B) with individuals colored according to provinces of Figure 1. **C)** For each province, the violin plots show how dispersed the individuals from that province are in panels A and B compared to a random set of similar size.


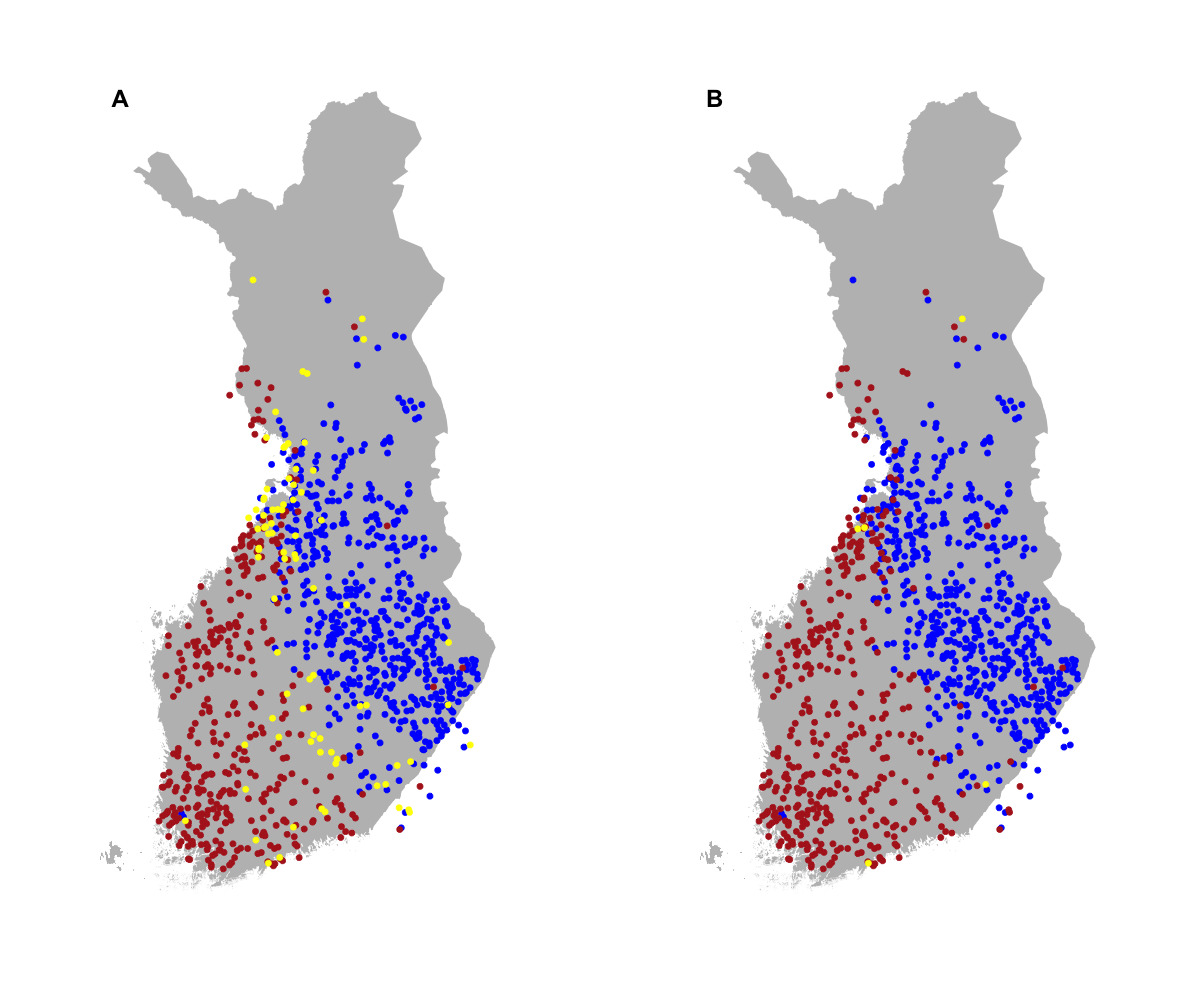


**Figure S2.** Uncertainty of FineSTRUCTURE’s binary population assignment of Fig. 3A. Panel A shows in yellow those (80) individuals whose probability in both populations is at least 1% and panel B those with probability of at least 20 % (5 individuals). Here probability is estimated from the raw population assignments over the MCMC runs. Panel B corresponds to the same level of uncertainty as our Gaussian mixture model (GMM) presented in Fig. 3B, which shows that FS is much more confident in its population assignments than our GMM approach. FS clustering algorithm is unsupervised whereas our GMM is supervised.


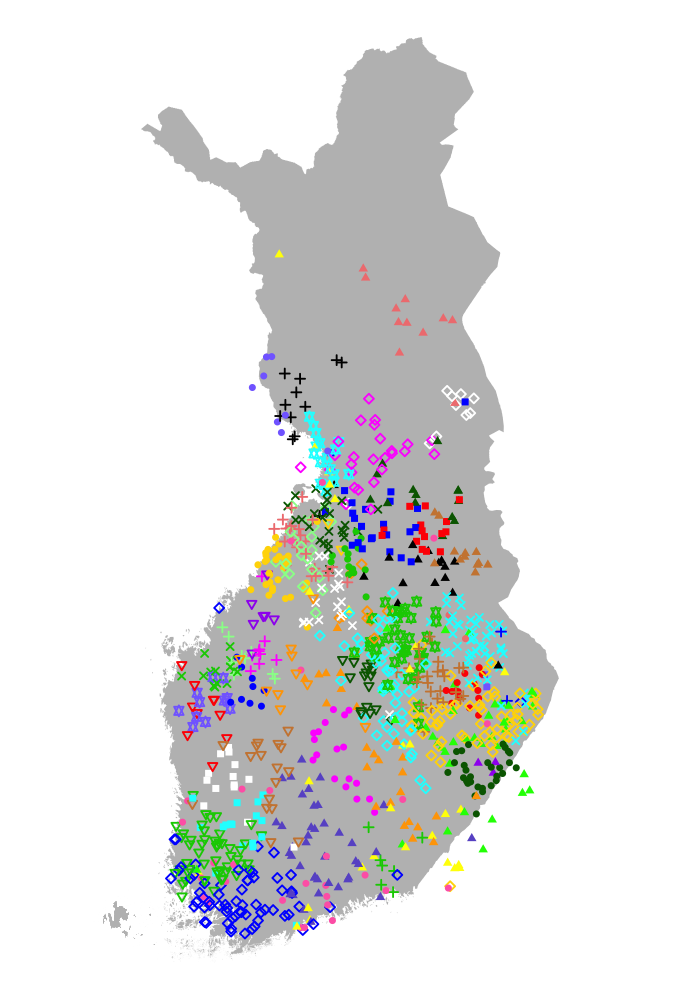

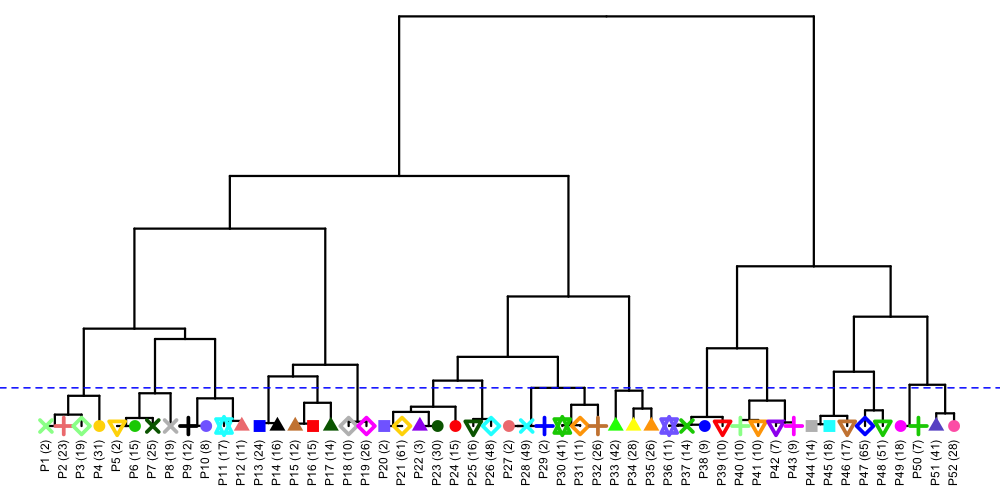

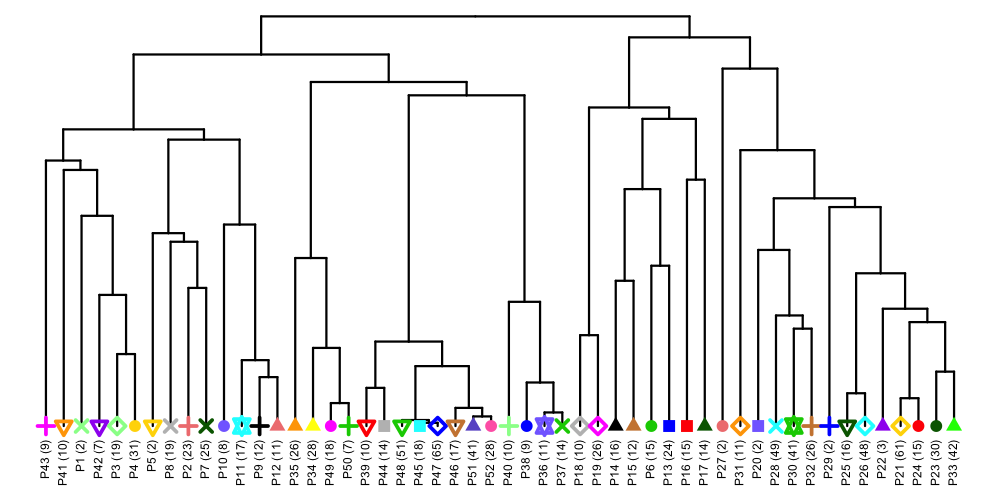


**A**

**B**

**C**

**Figure S3.** All 52 genetic populations in the data set of 1042 individuals (panel **A**) and their relationships according to FS-tree (**B**) and to TVD-tree (**C**). The horizontal line in panel B denotes the level of 17 populations shown in Fig. 4. The number of individuals in each population is listed in parentheses.

**
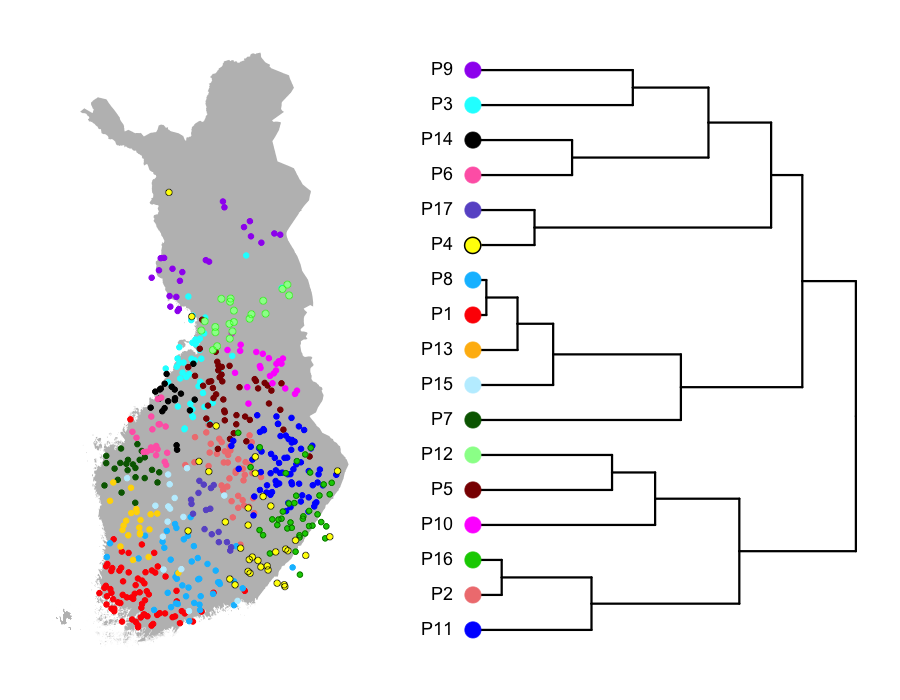
**

**Figure S4.** Population structure of 580 individuals at level 17 and the corresponding TVD-tree. Compare to Fig. 4A.

**
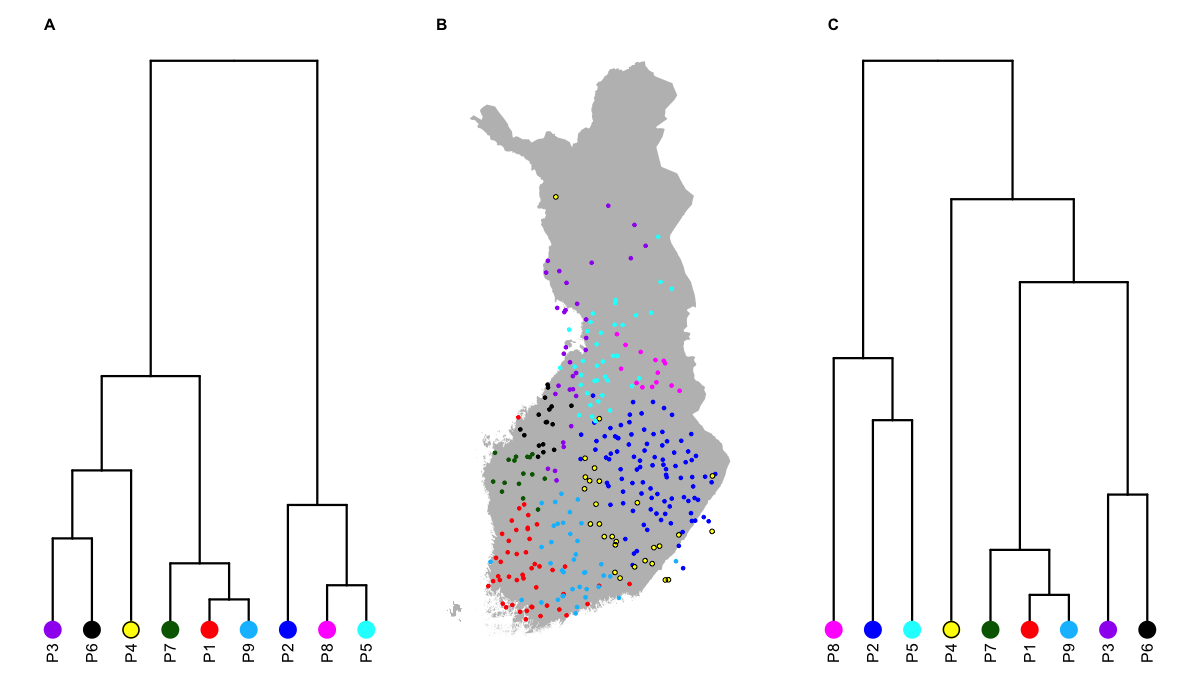
**

**Figure S5.** Comparison of FS-tree (A) and TVD-tree (C) using 328 individuals at the level of 9 populations. Panel B shows the population assignment of each individual on the map of Finland.


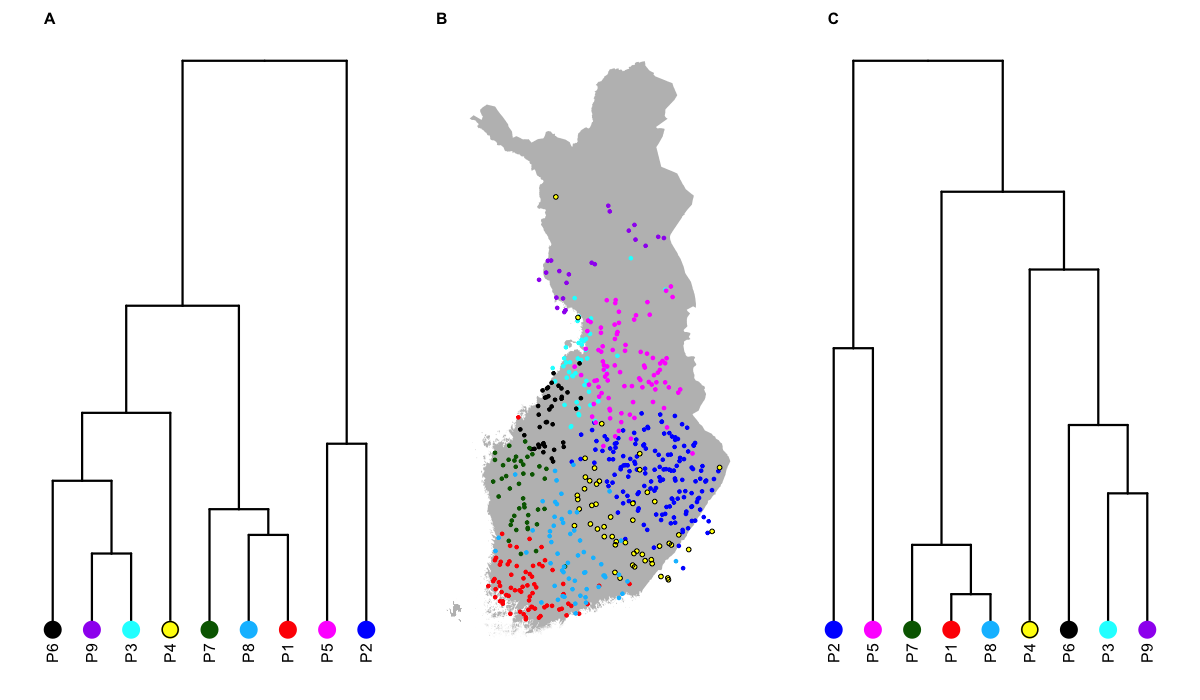


**Figure S6.** Comparison of FS-tree (A) and TVD-tree (C) using 580 individuals at the level of 9 populations. Panel B shows the population assignment of each individual on the map of Finland.

**
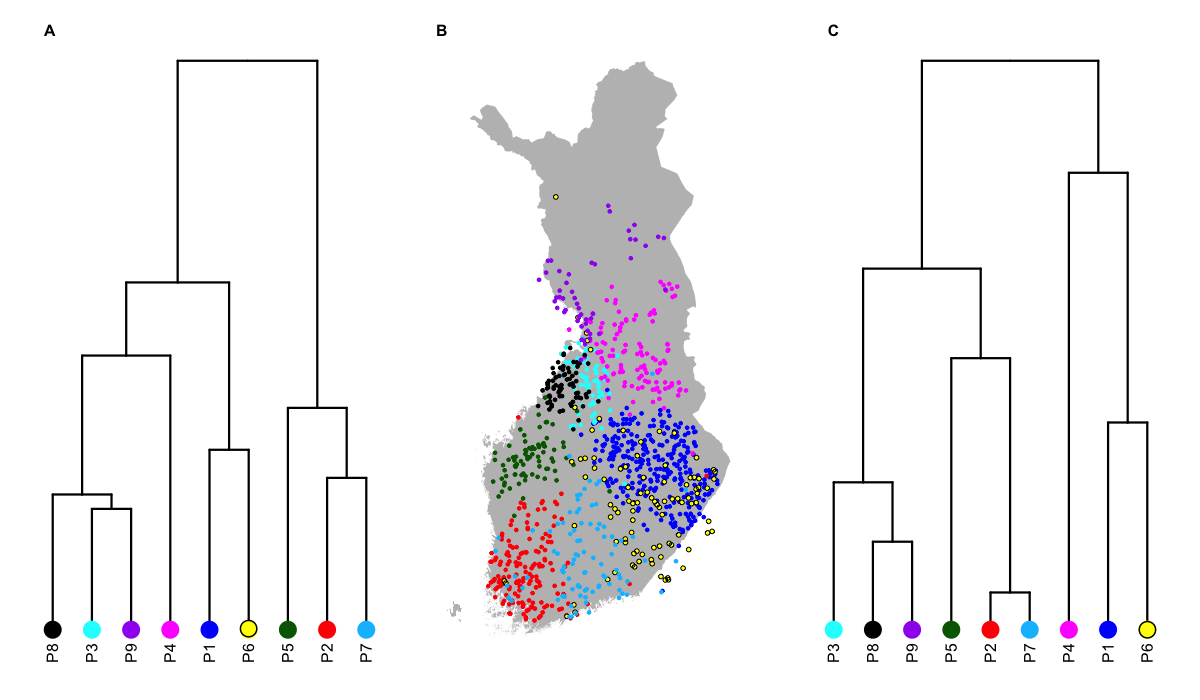
**

**Figure S7.** Comparison of FS-tree (A) and TVD-tree (C) using 1042 individuals at the level of 9 populations. Panel B shows the population assignment of each individual on the map of Finland.

**
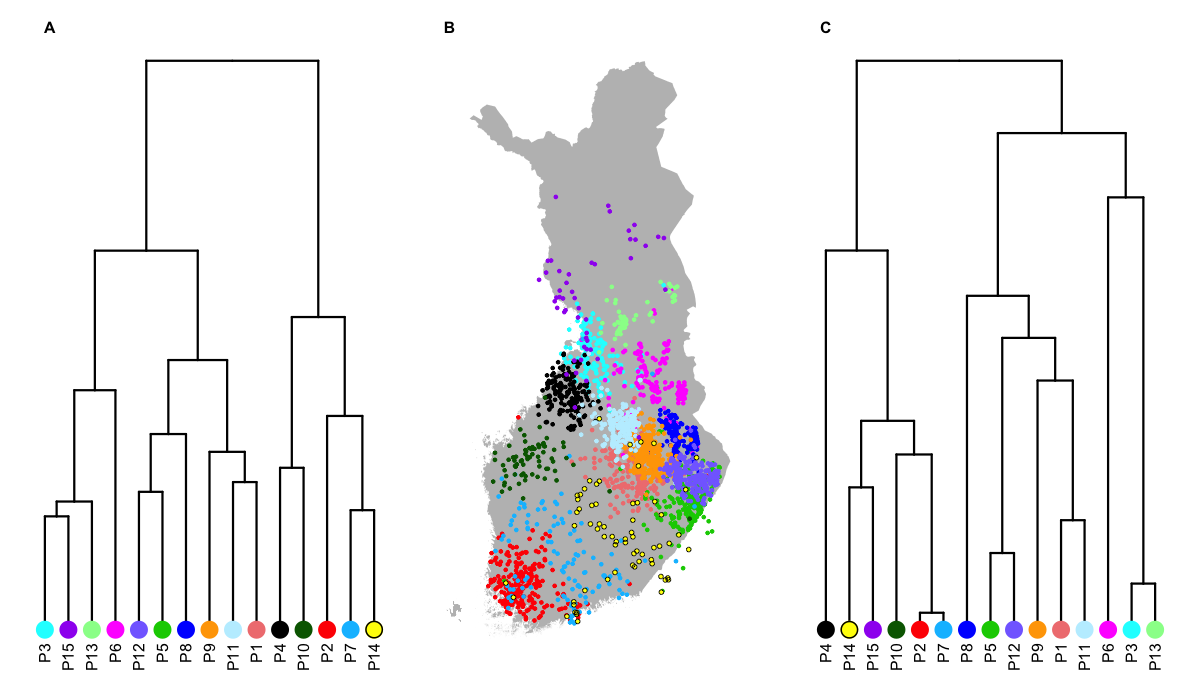
**

**Figure S8.** Comparison of FS-tree (A) and TVD-tree (C) using 2376 individuals at the level of 15 populations. Panel B shows the population assignment of each individual on the map of Finland.


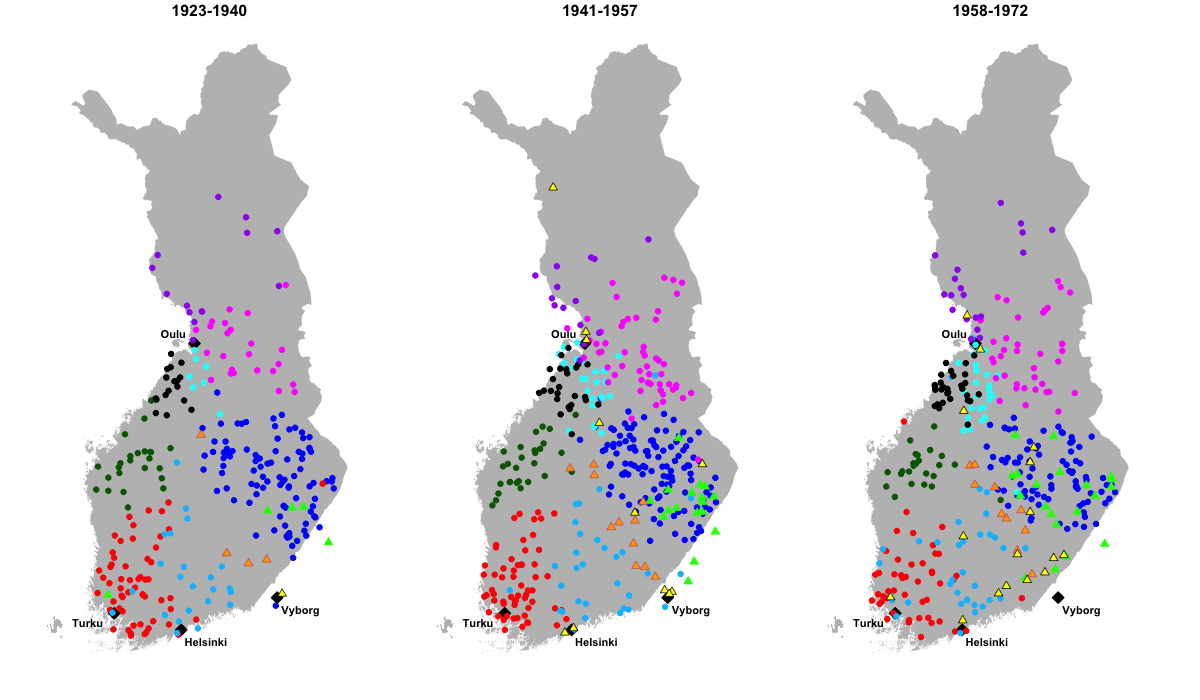


**Figure S9**. Populations by birth year of the samples. The eight populations marked with round symbols are from Fig. 5B and three populations marked with triangles represent subpopulations of P6 of Fig. 4. (Population P6 also matches exactly with the yellow population in Fig. 5B.) We see that the geographical locations of the eight populations do not noticeably change over time but two of the three subpopulation of population P6 seem to spread out over time, namely the subpopulations marked with yellow and green triangles.


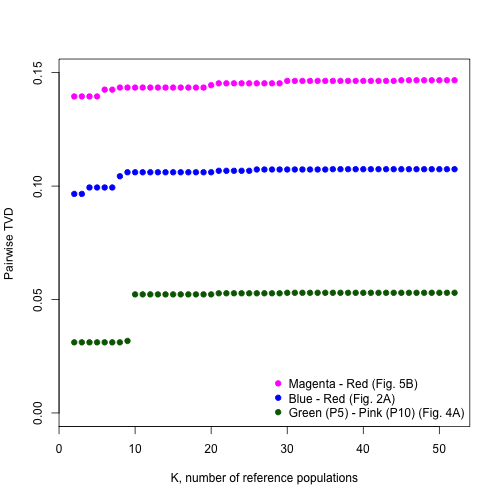


**Figure S10**. The effect of the number of reference populations (K) on the pairwise total variation distance (TVD) for three different pairs of populations. The pairs represent close (green), medium (blue) and distant (magenta) relatedness and, for all pairs, pairwise TVD seems robust to K when K > 10.
